# Influences of landscape change and winter severity on invasive ungulate persistence in the Nearctic boreal forest

**DOI:** 10.1038/s41598-020-65385-3

**Published:** 2020-05-26

**Authors:** Jason T. Fisher, A. Cole Burton, Luke Nolan, Laurence Roy

**Affiliations:** 1 University of Victoria, School of Environmental Studies, Victoria, British, Columbia, Canada; 2 Former address: InnoTech Alberta, Bag 4000, Vegreville, Alberta, T9C1T4 Canada; 3 University of British Columbia, Department of Forest Resources Management, Forest Sciences Centre, 2045 – 2424 Main Mall, Vancouver, British, Columbia, V6T1Z4 Canada

**Keywords:** Boreal ecology, Conservation biology, Invasive species

## Abstract

Climate and landscape change are drivers of species range shifts and biodiversity loss; understanding how they facilitate and sustain invasions has been empirically challenging. Winter severity is decreasing with climate change and is a predicted mechanism of contemporary and future range shifts. For example, white-tailed deer (*Odocoileus virginianus*) expansion is a continental phenomenon across the Nearctic with ecological consequences for entire biotic communities. We capitalized on recent temporal variation in winter severity to examine spatial and temporal dynamics of invasive deer distribution in the Nearctic boreal forest. We hypothesized deer distribution would decrease in severe winters reflecting historical climate constraints, and remain more static in moderate winters reflecting recent climate. Further, we predicted that regardless of winter severity, deer distribution would persist and be best explained by early seral forage subsidies from extensive landscape change *via* resource extraction. We applied dynamic occupancy models in time, and species distribution models in space, to data from 62 camera traps sampled over 3 years in northeastern Alberta, Canada. Deer distribution shrank more markedly in severe winters but rebounded each spring regardless of winter severity. Deer distribution was best explained by anthropogenic landscape features assumed to provide early seral vegetation subsidy, accounting for natural landcover. We conclude that deer dynamics in the northern boreal forest are influenced both by landscape change across space and winter severity through time, the latter expected to further decrease with climate change. We contend that the combined influence of these two drivers is likely pervasive for many species, with changing resources offsetting or augmenting physiological limitations.

## Introduction

The twin spectres of climate change and landscape change loom behind the human domination of global ecosystems and biodiversity loss in the Anthropocene^1,2^. Climate change and landscape change likely interact to affect biotic communities^3,4^, but interactions are difficult to quantify, as they can be additive, antagonistic, or synergistic^5^. Moreover, species distributions vary in time and space with habitat availability, community assembly, abiotic and climatic variables, and a host of other factors. Identifying the main drivers of a species’ distribution is the goal of niche ecology^6^ and a requisite for conservation plans to curtail biodiversity loss, so understanding the relative contributions of landscape and weather variability to changes in population size and distribution is a key ecological pursuit.

For mammals, responses to climate – and the weather it manifests over small temporal scales – are often rooted in changing energetic demands^7,8^. Likewise, mammal responses to landscape change can trace proximal causes to metabolic costs^9^. Direct habitat loss and isolation incurred by habitat fragmentation are two commonly cited mechanisms of biodiversity loss^10,11^, but disturbing established vegetation communities can create resource subsidies for some species – a notable mechanism of species invasions^12^. In the North American boreal forest, for example, anthropogenic disturbance replaces mature forest with early seral vegetation that benefits some mammal species over others^13,14^. Balancing energy intake and metabolic demands is particularly important for boreal species, where winters can be severe and food supplies ephemeral^15,16^. The interplay between landscape change and severe weather should therefore be expected to be particularly important in this cold, energetically costly, nutritionally poor biome.

We examined the roles of landscape change – defined here as anthropogenic landscape features – and variable winter severity in distribution dynamics of invasive white-tailed deer (*Odocoileus virginianus*, hereafter “deer”) in the western Canadian boreal forest. Deer have expanded markedly across the continent since European colonization^17^, affecting forest structure, community composition, biodiversity, ecosystem dynamics, and predator-prey dynamics^18^. In the vast northern boreal forests, deer expansion has resulted in woodland caribou (*Rangifer tarandus*) declines^19,20^ through apparent competition^21,22^. The alarming and ongoing caribou declines^23^ and expected suite of ancillary effects of deer invasion^24^ prompt a need to understand the ecological factors sustaining deer invasion.

As ruminants white-tailed deer rely greatly on sufficient nutritious forage^25,26^. In mature boreal forests early seral vegetation is limited to regenerating disturbances from fire, insects, and canopy caps^13^. Thus, deer metabolic demands may have eclipsed energy derived limited forage, likely restricting deer distribution from the boreal^25,27,28^. In winter when snow is deep deer incur great metabolic demands from movement stressing them energetically; they experience starvation, and pre-season stores and available forage control this starvation, otherwise mortality ensues^25^. With ongoing climate change, winters globally have become less severe^29^, and evidence suggests this is true of boreal regions as well, with less snow (Fig. S1) and less extreme temperatures^30^. At the same time the landscape footprint of forest harvesting, energy exploration and extraction, and transportation infrastructure has generated a spatially extensive array of early seral vegetation, smaller in patch size but much more widely dispersed, and greater in overall area, than natural disturbance regimes^31,32^.

In the boreal forests of Alberta, Canada, provincial-scale aerial deer survey and snowtracking data modelled with remotely sensed landcover data suggest climate change drives white-tailed deer invasion whereas landscape change plays a secondary role^27,33^. However, processes notoriously differ across scales^34,35^, and the landscape-scale processes facilitating invasion remain unknown. Empirical estimation of landscape-scale deer distribution dynamics across winters of differing severity, and of deer spatial distribution relative to anthropogenic footprint, would greatly help elucidate the relative influence of landscape and climate change on deer invasion.

To that end we examined deer spatiotemporal dynamics in Alberta’s northeastern boreal forest using camera trapping – an increasingly popular ecological research tool^36,37^ – analyzed using spatially explicit, hierarchical models for repeat occurrence data^38^, and species distribution models^39^. Climate change is a long-term trend, thus responses to climate change are concomitantly lengthy to document. In the immediate term, response to extreme weather events^40^ that mirror historical and contemporary climate is a useful proxy for predicted climate change effects on biota^41,42^. We capitalized upon marked variation in boreal winter weather severity over three years, representative of historically severe and contemporary moderate winters (Fig. S1), to test two hypotheses: (1) Deer distribution fluctuates in time, contracting markedly in response to in severe winters typical of 20^th^-century climate, but rebounds in spring, leaving relatively stable annual distributions; (2) Deer spatial distribution is positively related to anthropogenic features creating early seral vegetation subsidies, both annually and in the winter season. Understanding the influences of both landscape disturbance and energetically costly severe weather in sustaining invasive populations is a critical endeavour, as these processes are expected to rapidly increase with global change.

## Materials and Methods

### Study area

**Figure 1:**
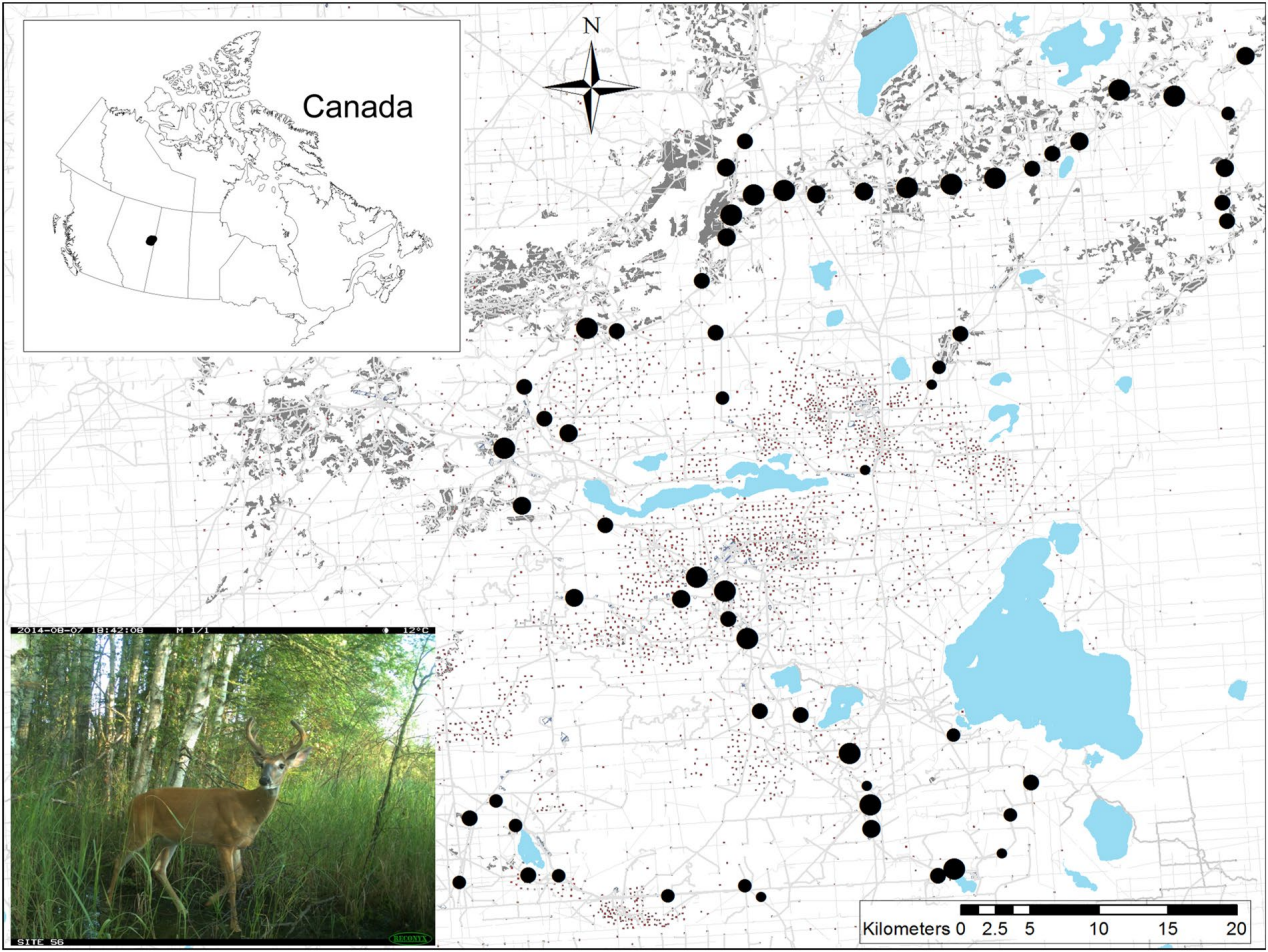
White-tailed deer occurrence was surveyed at 62 camera sites (large block dots, scaled to the relative number of observations over three years) in the boreal forest of northeast Alberta, Canada. Anthropogenic footprint is extensively and intensively imposed on this landscape, including forest harvesting cutblocks (grey polygons), well sites (square dots), seismic lines (grey), and roads and trails (dark grey and colored lines). Lakes are in blue. Map was created in ArcGIS v.10 based on Alberta Biodiversity Monitoring Institute Human Footprint data 2010 (abmi.ca) and Government of Alberta water bodies data.

We surveyed white-tailed deer distribution in northeast, Alberta, Canada (Fig. 1), within a boreal forest ecosystem invaded by northward-expanding deer populations in the last few decades. The 3000 km^2^ study area is a mosaic of white (*Picea glauca*) and black spruce (*Picea mariana*), aspen (*Populus tremulodies*), jack pine (*Pinus banksiana*), and *Ledum groenlandicum*-dominated muskeg. Extensive petroleum exploration and extraction features, forest harvesting, roads (car accessible), off-highway vehicle (OHV) trails, and other landscape disturbances are dispersed throughout the study area (Fig. 1).

**Figure 2:**
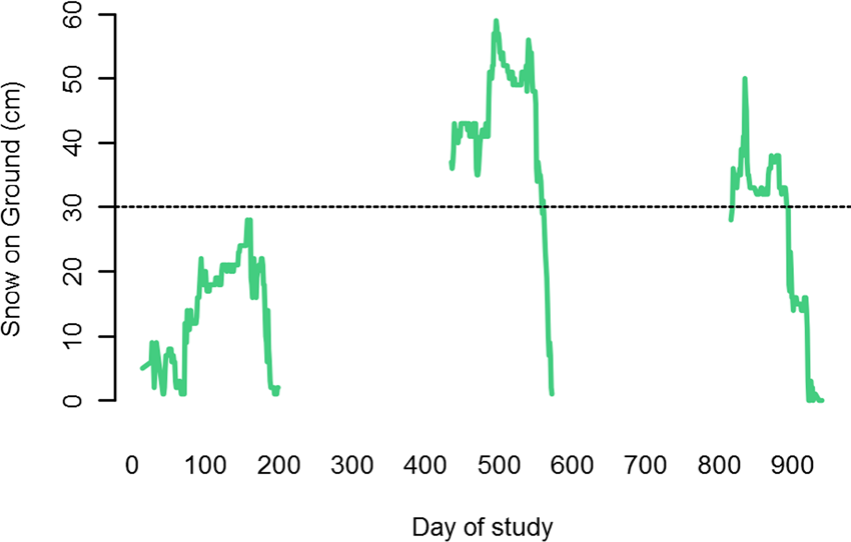
Winter severity as indicated by snow-on-ground, from Environment Canada weather data, Fort McMurray, Alberta, 2011–2014, summarised daily. The 30-year mean maximum snow depth (dashed line) is 30 cm. The winter of 2011–12 exhibited contemporary average snow depth, whereas the winters of 2012–13 and 2013–14 were comparatively severe.

We used Environment Canada weather data (http://climate.weather.gc.ca) for Fort McMurray, Alberta–the closest station with complete data– to measure winter severity in the study region. White-tailed deer are highly susceptible to snow depth, which incurs a substantial metabolic cost leading to overwinter mortality^26,43–45^, so we used daily mean snow depth (“snow-on-ground”) for the region across the three years of study (Fig. 2). Fluctuations across this period provided a severe snowfall winter typical historically (Fig. S1), a moderate snowfall winter typical of contemporary winters, and one between these extremes.

### Deer distribution in space and time

We sampled deer occurrence at 62 camera trap sites deployed in a constrained stratified random design^46^ based on digital forest inventory data, as in^14^. In a geographic information system (GIS) we reclassified the landscape into landcover categories (Table S1) and overlaid it with 1 × 1-km grid cells. We categorized each grid cell by the most abundant landcover within it, then randomly selected grid cells as sampling sites, such that each category received equal sampling effort. Random selection was constrained to induce a minimum 2-km distance from other sites to maintain independence, and multiple different individuals were indeed identified at adjacent sites. The resulting design produced some sampling site clustering (Fig. 1) induced by the naturally clumped distribution of landcover classes on the landscape, but captured the range of this landscape’s anthropogenic footprint and natural heterogeneity. Cameras were placed on active wildlife trails embedded in forests, a minimum of 200 m from the roads and trails used to access the grid cell. The area is so replete with these linear features (Fig. 1) that although roads and trails are more abundant within 250-m of our sites than within the study landscape, this effect falls away at 500-m radius of quantification, and at the 1-km radius scale of our analysis (Fig. S2) roads are represented in our sample as they exist in the landscape. We obtained permission from government land officers and industry leaseholders to access all sampling areas. We deployed one Reconyx PC900 Hyperfire infra-red remote digital camera (Holmen, WI, USA) at each site for three years: October 2011 - October 2014. We define ‘site’ as the area likely to be used by a deer within a three-month season, centered on the camera detection zone. We assume the study area is the *ca*. 3000 km^2^ area encompassing a minimum convex polygon surrounding the camera sites.

### Modelling deer occupancy dynamics through time

Modelling serial occurrence data, such as repeated detections *via* camera traps, is an area of active research without current consensus, and there is much debate about how well many species distribution models actually meet their assumptions^36,47–49^. To analyze deer distribution across time we used hierarchical occupancy models^50^ which can be considered as simultaneous generalized linear models (GLMs) of serial detection data, applied to each component of the model, with binomial errors (logistic link). In this model we consider zeroes in serial detection histories (e.g. 1010) as resulting from movement of this vagile animal into and out of the site^49,51,52^; Variation in *p* – the probability of detecting that species if present – is attributed mainly to movement in and out of the camera detection zone^36,51,52^; this is important since deer movement rates are likely to vary seasonally, particularly with snow cover. With variance due to *p* thus partitioned, we estimate occupancy as the probability of site use within each season. With multi-season models, species distributions can be considered closed during a survey and open to change among them; occupancy dynamics can be modeled through time, providing estimates of local site “extinction” and colonization^53^. Probabilities of occupancy (*ψ*), local extinction (ε), and colonization (γ) are estimated *via* a first-order Markov process in a maximum likelihood framework wherein the probability of occupancy at a site in year *t* + 1 is contingent on occupancy in year *t*, and:

ψ_1_ = probability a site is occupied in year 1

ε_*t*_ = probability an occupied site becomes unoccupied between seasons *t* and *t* + 1

γ_*t*_ = probability an unoccupied site becomes occupied between seasons *t* and *t* + 1

λ_*t*_ = spatial growth rate or rate of change in occupancy, ψ_*t+1*_ / ψ_*t*_

*p*_*t,j*_ = probability that deer are detected at a site in survey *j* of season *t* (given presence)

We used multi-season occupancy models to estimate the change in deer distribution among seasons spanning three years. We separated continuous camera data into month-long (30.4 day) “secondary” survey periods^53^. Three secondary surveys comprised a primary sampling season. Deer behaviour varies seasonally, with most variation due to changes associated with mating, parturition, and dispersal^54^. We therefore classed each “deer season” as three-month periods: rut (autumn, October – December), post-rut (winter, January – March), pre/early fawning (spring, April – June), and post-fawning (summer, July – September). We relax the closure assumption and assume non-Markovian variation in deer site-use among months within a 3-month season primary season^38^. The full data frame for the study is thus 12 seasons, with 3 repeated surveys within each season, for a total of 36 surveys at each site, each comprised of deer detection-nondetection within the month.

We ran several competing occupancy models in software *Presence*^55^. We tested whether *p* was constant or varied among seasons or surveys; whether occupancy (*ψ*), site colonization (γ), and site extinction (ε) were either constant or varied among seasons. We sought to estimate mean differences in *ψ* among seasons across the study area, and moreover models incorporating landcover classes (*q.v*.) as covariates of *ψ*, γ, and ε did not converge due to the data demands of occupancy models and problems with border estimates; occupancy and recolonization often approached 1.0. Therefore we assumed spatial homogeneity in *ψ*, which should then be interpreted as a seasonal landscape average of the proportion of the area occupied by deer. We ranked competing models *via* Akaike’s Information Criterion (AIC) in an information-theoretic approach, normalised them as AIC weights (AIC_w_), and calculated evidence ratios (ER) for each variable^56^. From per-survey estimates of *p* we calculated the probability of false absence (PFA) within a season as [1-*p*]^3,57^.

### Modelling deer distribution across space

Species distribution models assume that the repeated occurrence or persistence of a species at a site is related to the environment within some defined area around that site^58^. As the variable use of a site by a species is often an ecological signal (rather than error) we modelled serial detections and nondetections across space to understand deer response to landscape change. As for the temporal analysis, our dependent data remained as above: the repeated occurrence of deer among 36, one-month survey periods, yielding a 0–36 response variable. Monthly detections serve to minimize effects of temporal heterogeneity in detection rates induced by using all detections, such as changes to detection rates caused by variable movement^36^. Each month can be considered an independent Bernoulli trial in which the deer was detected (1) or not (0) at a site^59^.

We quantified the study area landscape using three spatial digital resource inventories. We quantified natural land cover within multiple buffers around each camera site using Alberta Vegetation Inventory (AVI), a digital forest inventory dataset. We quantified the percent of area of polygonal anthropogenic features in these buffers from the Alberta Biodiversity Monitoring Institute (ABMI) 2010 Human Footprint Map Ver 1.1 (ambi.ca), and ABMI linear features layer derived from 2012 SPOT satellite imagery (Table S1). We omitted correlated variables (r > 0.7)^60^ to prevent multicollinearity. These data were the most contemporary available to match our deer sample available; we did not expect the landscape to change extensively during the 1–2 years separating the datasets. We combined sparsely represented variables (<1–2% of area) into a single, combination variable (Table S1). We rescaled each variable (mean = 0, s.d.=1) to compare effect sizes.

It is challenging to identify the area of habitat around each sampling site that best explains species occurrence (*spatial scale*), as different processes operate at different spatial scales^35,61^. One approach is to quantify habitat at multiple spatial scales, run multivariate species distributions models at each scale, and determine the spatial scale at which habitat best explains species occurrence^14,62^. We modelled the 0–36 months of white-tailed deer occurrence against landscape variables quantified at 20 spatial scales ranging from 250-m to 5000-m radius around each camera site. Each generalized linear model (binomial errors, logit link) in *R* ver. 3.2.2 (R Foundation for Statistical Computing 2014) regressed deer persistence against all 20 landscape variables (Table S1). We used the *step-AIC* function in R package *MASS*; to identify the spatial scale that generated the best-supported model explaining deer occurrence^63^, plotted as normalised AIC_w_^56,64^.

We used model selection to weigh support for 30 competing, non-mutually exclusive hypotheses about deer response to natural and anthropogenic landscape features (Table S2). These hypotheses were grouped into candidate sets, with each set assuming deer distribution was driven by (1) natural landcover, (2) nonforested areas, (3) forestry features; (4) petroleum extraction features; (5) petroleum and forestry combined; (6) human access linear features; or (7) combinations of natural features and (3–6). Each hypothesis was represented by a generalised linear model (binomial errors, logit link), and generated an AIC score and normalised AIC_w_, which we ranked to identify the best-supported model among the candidate sets. We tested the best supported model for overdispersion, and calculated the deviance explained by each model^65^. We used R package *boot*^66^ to assess model fit with 10-fold cross validation^67^, and calculated Moran’s I to test for spatial autocorrelation of model residuals.

This approach allowed us to test hypotheses about annual deer occurrence. However as winter is critical for deer survival^25,68^, and winter severity is expected to change with climate change, we also asked whether the same landscape factors influenced deer occurrence within winter periods only, repeating this analysis with data from winter months (January-March) yielding a 0–9 response variable (number of winter months over three years in which deer occurred at a site).

### Research ethics

All research was permitted by the Government of Alberta, Ministry of Environment and Parks, Fish & Wildlife Division, Collection License 49143.

### Animal ethics

This research was reviewed and approved by InnoTech Alberta’s Animal Care and Use Committee (ACUC), permit ACUC0524.frm /clj/IO.II.02. No animals were handled in this phase of the study.

## Results

White-tailed deer were identified in 112,648 of 141,140 animal images captured by the camera trap survey, spread across the study area, and are now by far the most prevalent large mammal in this system (Fig. 1). Winter severity was markedly different among the three years (Fig. 2). The 30-year mean annual snow depth (snow on ground) –measured in February, when snow is deepest –at the Fort McMurray weather station is 30 cm^69^. Snow depth exceeded 30 cm in 0 days in 2011–2012; 125 days in 2012–2013; and 73 days in 2013–2014. We interpreted these as mild, severe, and moderately severe winters, corresponding to snow-on-ground variability over the last century (Fig. S1).

### Deer occupancy dynamics through time

**Figure 3:**
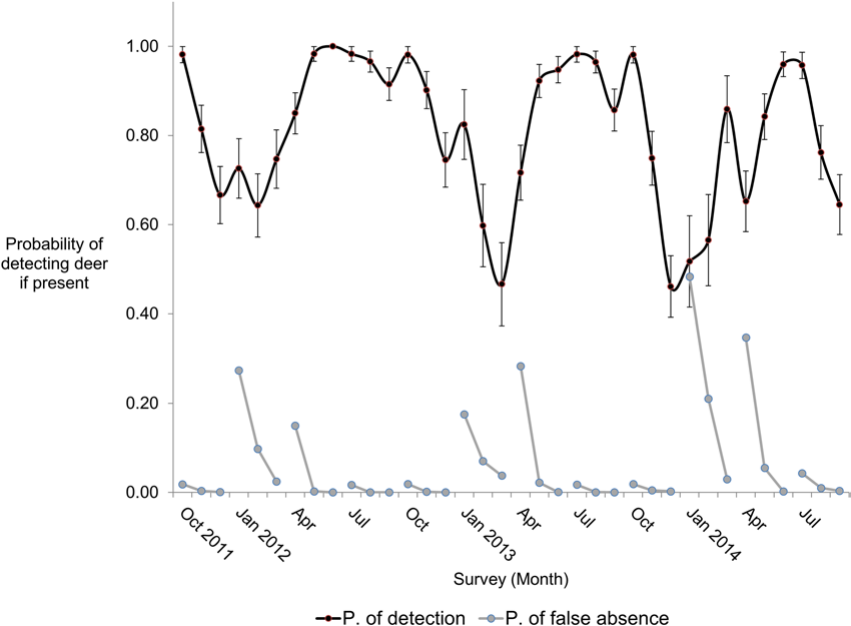
The probability of detecting deer via cameras (black line) varied monthly. The probability of false absence (blue lines) within each season approached 0.0 for most seasons and at worst (spring 2014) was 3%. Bars represent standard errors of estimates.

The probability of deer site extinction and colonization varied seasonally, and the probability of detecting deer given their occurrence at a site (*p*) varied monthly; this model carried 100% of the AIC weight among the set of competing candidate models of detectability (Table S3). Estimated *p* was lowest in late winter and spring and highest in summer (Fig. 3), providing support for the prediction that deer yard in winter and *p*, being affected by movement, reflects this lower movement rate. PFA approached 0 in most seasons and at worst was 0.03, indicating that deer were detected at sites where they occurred and lending confidence to our estimates of occupancy.

**Figure 4:**
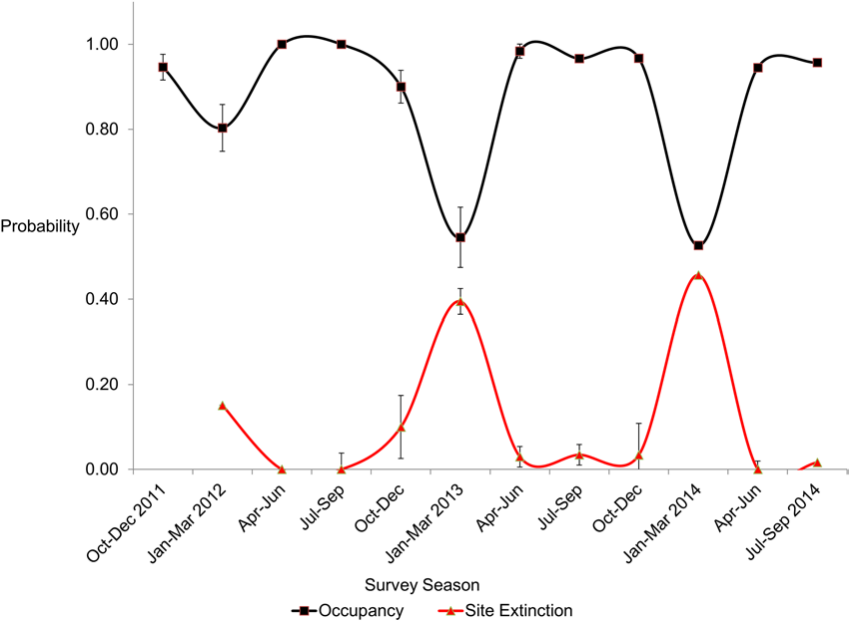
The probability of white-tailed deer occupancy (black line) and the probability of site extinction (red line) varied among seasons. Site extinction was greatest in the two severe winters.

Deer occupancy fluctuated widely among seasons (Fig. 4). Deer occupied nearly the entire study area at the onset of autumn 2011. Occupancy dropped slightly in the first mild winter, then rebounded in the following spring. Occupancy remained mostly high and stable through the summer and autumn, and then dropped precipitously in the severe second winter of 2012. Deer occupancy again rebounded in spring 2013. This same pattern repeated through the third, moderately severe winter of 2013–2014 (Fig. 4).

**Figure 5:**
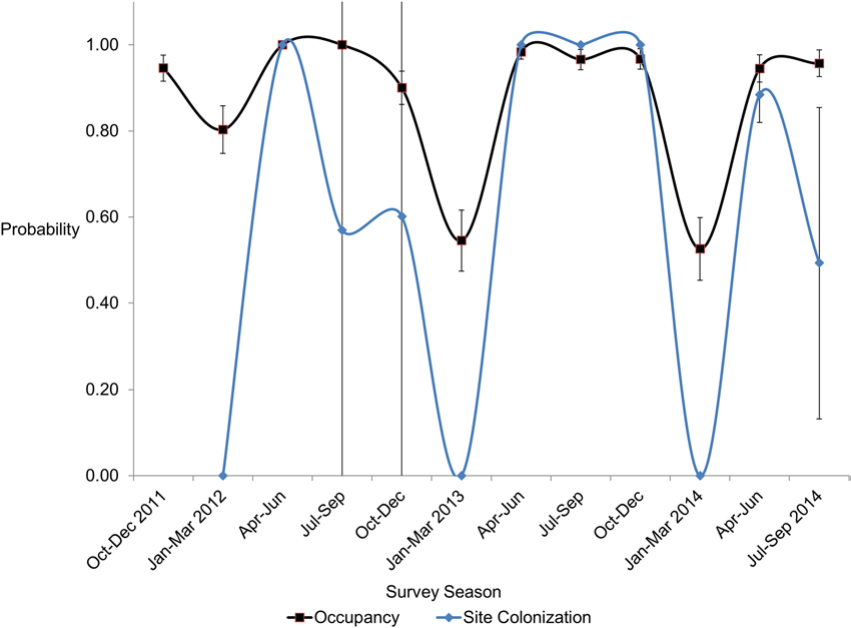
The probability of white-tailed deer occupancy (black line) and site colonization (blue line) varied among seasons and was high in all three spring periods. Bars represent standard errors.

These occupancy dynamics were driven (in part) by the probability of site extinction (ε) (Fig. 4). Estimated ε was low (0.15, SE = 0.05) in the mild 2012 winter, but greater in the severe 2013 winter (ε = 0.39, SE = 0.07). About 40% of sites were emptied of deer in the winter, and this dynamic repeated in the third year (ε = 0.46, SE = 0.07). Occupancy dynamics were also driven by the probability of site colonization (γ) (Fig. 5). After the 2012 mild winter, all empty sites were recolonized (γ = 1.0, SE = 0.00). After the severe 2013 winter, the landscape was again fully recolonized (γ = 1.0, SE = 0.00); after the moderately severe 2014 winter, site recolonization was again very high (γ = 0.88, SE = 0.06). Colonization was difficult to estimate in summer and fall because most sites were occupied; standard errors were large due to difficulties in model convergence and parameter estimation at border conditions (*e.g*. 1 or 0).

**Figure 6:**
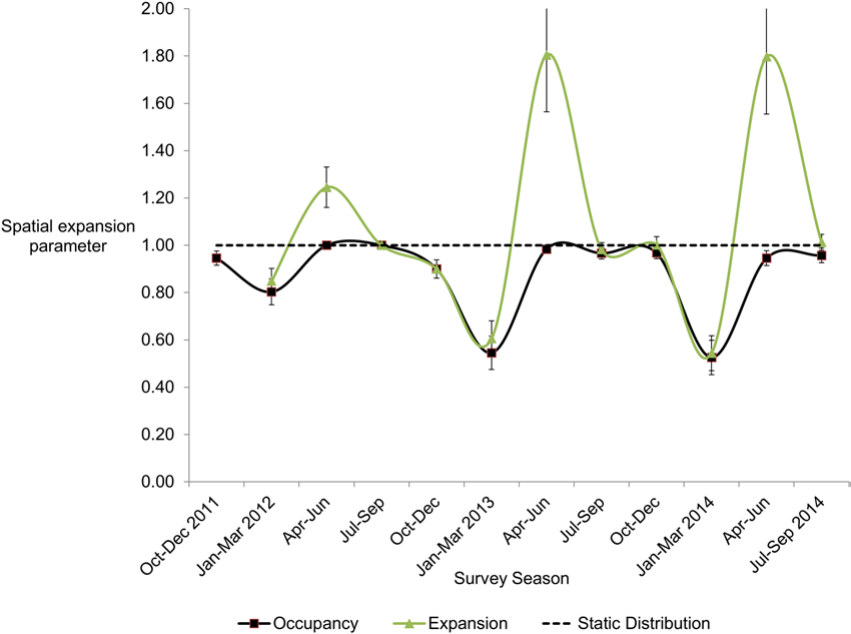
The spatial expansion parameter of boreal white-tailed deer varied among seasons and was >1 in all three spring periods. Bars represent standard errors of estimates; the dashed line represents zero spatial growth and a stable distribution.

Distribution dynamics can be synthesized as a spatial expansion parameter or “spatial growth rate” (λ), analogous to population growth rate^53^. Distribution is stable if λ = 1.0, shrinking if λ <1, and expanding if λ> 1^53^. Deer λ declined more markedly in the severe winters of 2013 (λ = 0.61; SE = 0.07) and 2015 (λ = 0.54, SE = 0.07) than the mild winter of 2012 (λ = 0.8, SE = 0.05) (Fig. 6). However, deer distribution rebounded dramatically in both subsequent springs (λ = 1.8, SE = 0.24), returning to a stable distribution (λ = 1.0, SE = 0.03) in each summer and fall season – despite winter severity.

### Deer distribution across space

Of the 20 different spatial scales we examined, landscape characteristics around a site measured at the 1000-m radius scale best explained deer site persistence both annually (AIC_w_ = 0.98) and for winter months (AIC_w_ = 0.90), supported by most of the weight of evidence (Fig. S2, S3). White-tailed deer annual persistence was best explained by model 28: upland deciduous forest and anthropogenic features (Table 1). However, not all the variables in this candidate model were significant (i.e. with β coefficients different from 0); as per^64^ we identified the most parsimonious model by *post hoc* dropping variables with p> 0.2. The resulting best-supported parsimonious model (AIC_w_ = 0.99) with significant β coefficients indicated deer distribution was explained by anthropogenic landscape features deriving from a combination of forest harvesting, oil and gas exploration and development, and transportation infrastructure. Industrial block features, petroleum extraction well sites, and forest harvest cutblocks had large positive effects on deer persistence (Table 1). Deer persistence was negatively associated with off-highway vehicle trails.

**Table 1:** Parameter estimates from the best-fit, most parsimonious generalized linear model (binomial errors, log link) of annual deer persistence (number of months of deer presence, out of 36), modelled against landscape features.

Parameter	β estimate	std. error	t-value	p-value
(Intercept)	−0.16	0.10	−1.60	0.1090
Upland deciduous forest	0.03	0.00	7.07	<0.0001
Forest harvest blocks	0.07	0.01	7.76	<0.0001
Petroleum well sites	0.24	0.04	5.55	<0.0001
All trails (unpaved roads)	−0.88	0.17	−5.24	<0.0001
Industrial block features	0.41	0.06	7.37	<0.0001

*Null deviance = 465.4 on 60 df; residual deviance = 191.26 on 55 df; AIC = 397.85.

The same relationships were observed in winter, where model 28 (Table S2) was the best supported of the a priori candidate set, which we reduced *post hoc* by dropping variables with *p* > 0.02 (Figs. S2, S3). This best supported parsimonious model (AIC_w_ = 0.97) suggested deer persistence in winter months was positively related to industrial block features, cutblocks, and well sites, and negatively related to trails (Table 2). Deer’s negative response to trails (β estimate) was twice as great in winter as for annual persistence.

**Table 2:** Parameter estimates from the best-fit generalized linear model (binomial errors, log link) of winter deer persistence (number of months of deer presence January-March over 3 winters, 0–9), modelled against landscape features.

Parameter	β estimate	std. error	t-value	p-value
(Intercept)	−1.44	0.22	−6.58	<0.0001
Upland deciduous forest	0.02	0.01	3.75	0.0002
Forest harvest blocks	0.11	0.02	6.30	<0.0001
Petroleum well sites	0.14	0.08	1.79	0.0734
All trails (unpaved roads)	−1.50	0.33	−4.58	<0.0001
Industrial block features	0.57	0.09	6.02	<0.0001

*Null deviance = 295.9 on 60 df; residual deviance = 160.27 on 55 df; AIC = 270.35.

All models fit the observed data relatively well; the best-fit model of annual deer persistence explained 58.9% of the deviance in observed deer detections, with a k-fold (k = 10) validation yielding prediction error of 1.9%. There was no significant spatial autocorrelation in model residuals (Moran’s I = 0.0393, expected = 0.0167, s.d. = 0.030, p = 0.062). The best-fit winter persistence model explained 45.8% of the deviance in the winter deer data and had a prediction error of 6.8%. The convergence of results across models suggests that a combination of upland deciduous forest and anthropogenic features – cutblocks, well sites, trails, and block features – best explained annual and winter white-tailed deer persistence.

## Discussion

Landscape change and decreased winter severity typical of contemporary climate change positively affect invasive white-tailed deer landscape-scale distribution in the western Nearctic boreal forest. Disentangling the relative importance of climate and weather on global biodiversity has been the focus of some debate^70^ and there may be synergies between the two^5^ that are crucial to understanding mechanisms of biodiversity change, such as biological invasions. Certainly a changing North American climate has led to less severe winters – including the boreal forest – with significant consequences expected for biotic communities^8,71–74^. We show that the distribution of invasive white-tailed deer did contract more steeply in severe winters – even after accounting for less movement in winter as deer yard up (as movement correlates with estimated *p* in occupancy models^49,75^). In severe winters with deep snow on ground, deer distribution dropped nearly 50%, whereas distribution declined only marginally in the mild winter with less snow on ground.

It is an important caveat that we studied only three years across differing weather conditions, and a long-term population analysis will better elucidate responses to climatic trends through time. Rather than wait decades however, because snow conditions varied so markedly across the years–mirroring both mild contemporary winters and severe historical winters–we contend our observations suggest a likely mechanism by which climate change affects deer distributions. Past research at provincial scales using aerial survey data showed winter severity, including snowfall, was a key driver of white-tailed deer invasions^27,33^. Our examination within a 3000-km^2^ landscape provides a higher-resolution estimation of seasonal dynamics in relation to snow, as well as response to specific anthropogenic landscape features. Historically, the metabolic costs of moving through deep boreal forest snow likely exceeded energy obtainable from limited available forage^25,45,76–78^. If metabolic costs exceeds fat stores and new forage deer die. If available forage provides fat stores and winter food to pat these costs, deer live through the winter. Overwinter deer mortality drives seasonal population cycles elsewhere^79^, but distribution and population in boreal landscapes remain unknown.

Crucially however, regardless of winter severity, deer distribution quickly rebounded each spring. After severe winters in which deer distribution shrank by half, they quickly recolonized almost all empty sites in spring. Photographic analysis showed adults in all sites in this season, indicating recolonization from overwinter refugia; the contribution of reproduction has yet to be examined. Nonetheless deers’ dynamic resiliency defies expectations that two severe winters would significantly decrease boreal deer distribution, and it remains unclear how many severe winters would achieve this reduction. We contend that the spatial association of deer with anthropogenic features implicates early seral vegetation subsidies as a driver of the spring rebound.

Deer persistence – annually, and in especially in winter – was positively related to anthropogenic features, as well as upland forest. The response to upland deciduous forest met predictions as white-tailed deer forage on leaves and stems of woody plants; conifers offer comparatively low nutritional value^25,26^. Anthropogenic features regenerate into young, early-successional deciduous plants providing abundant deer forage^13^. This region of the boreal forest has been heavily impacted by resource extraction from multiple sectors, including forest harvesting, transportation, and petroleum extraction – which creates seismic exploration lines, pipelines, well sites, and industrial block features^14^. Multiple resource sectors in this boreal landscape create a cumulative disturbance of a magnitude and pattern without historic analogue^31,32^. Each feature removes old or mature forest and resets successional trajectories, collectively increasing the availability of early seral vegetation and providing abundant deer browse. These landscape features are spatially associated with the persistence of white-tailed deer in the boreal landscape.

Linear anthropogenic features (OHV trails) were negatively associated with deer distribution, with almost double the effect size in winter. Linear features expedite travel by wolves^80^ especially in winter, thereby increasing predation rates on woodland caribou^81,82^, and we hypothesized the same for deer. We posit deer avoid landscapes with higher densities of these features due to mortality risk. Trails also permit motorized human access, and deer are harvested in this landscape. Recreational access can have a significant effect on ungulate distribution^83,84^ but surprisingly little research has been devoted to understanding the effect of industrial access on wildlife distribution^85^.

Across their vast species range, white-tailed deer habitat relationships vary among seasons^54,79^ and we expected the same in the boreal, with different selection in winter vs. annually. However, selection in winter and annually was concordant, suggesting factors driving boreal deer distribution are consistent: selection of deciduous and early seral forage, and avoidance of anthropogenic linear features. In comparison, provincial-scale back-casting models suggests climate change eclipses landscape change in importance to boreal deer expansion^33^. The apparent difference between that study and this might be due to assumptions inherent in respective modelling approaches; spatial scale-dependency^34,62^ from the provincial scale to the landscape scale of our study; or less likely, a change in mechanisms over the last few years. Both views provide insights. Landscape-scale distribution dynamics observed in this study are consistent with the hypothesis that climate and landscape change both support persistence of this invasive species, much as they are synergistic for so many other ecological processes^86,87^.

Finally, we show how a single camera-trapping array camera can be used to investigate both spatial and temporal mammalian distribution patterns through time, generating insights about the ecological effects of landscape and climate change. We repeat recent appeals for a global network of camera trapping arrays^37^ to collect biodiversity data across all of Earth’s ecosystems to inform conservation decisions in this era of rapid biodiversity loss.

## Conclusions

Mild winters typical of contemporary weather stemming from climate change support persistence of white-tailed deer in Nearctic boreal forests, but even severe winters do not suppress landscape-scale distribution of this invasive ungulate into spring. The positive association between deer persistence and anthropogenic features suggests that resource extraction is supporting white-tailed deer, providing nutritional subsidies that stabilize deer distributions despite fluctuation in winter severity. The expansion of white-tailed deer has been an ongoing continental-scale problem for over a century^18,28^, leading to species at risk declines in this system, and community changes across the Nearctic. Expansion has occurred despite heavy hunting and a massive increase in urban and rural residential landscapes. The conversion of mature forest, especially conifer forest, into deciduous and early seral vegetation is a primary mechanism of continental expansion. Our research suggests that anthropogenic disturbance stemming from multiple forms of resource extraction supports continued white-tailed deer persistence near this northern range limit. We conclude that mild weather resulting from climate change and landscape change operating in tandem have sustained invasions. Emerging research on other species and systems^86–88^ is likewise showing that the twin drivers of concurrent landscape and climate change are shifting physical conditions and resource availability, collectively altering species ranges, interactions, and ultimately biodiversity.

## Supplementary information

Supplementary information.

## Data Availability

All data and code used in this research have been uploaded as supplementary material.
