# Supplementary material for: Influences of landscape change and winter severity on invasive ungulate persistence in the Nearctic boreal forest
Source: Sci Rep. 2020 May 26;10:8742. doi: 10.1038/s41598-020-65385-3 (PMC7250834; doi:10.1038/s41598-020-65385-3)
Supplement: Supplementary file 1 — Supplementary information. [file 41598_2020_65385_MOESM1_ESM.docx]

Influences of climate and landscape change on invasive ungulate persistence in the Nearctic boreal forest.

Jason T Fisher, A. Cole Burton, Luke Nolan, and Laurence Roy

Supplementary Information

**
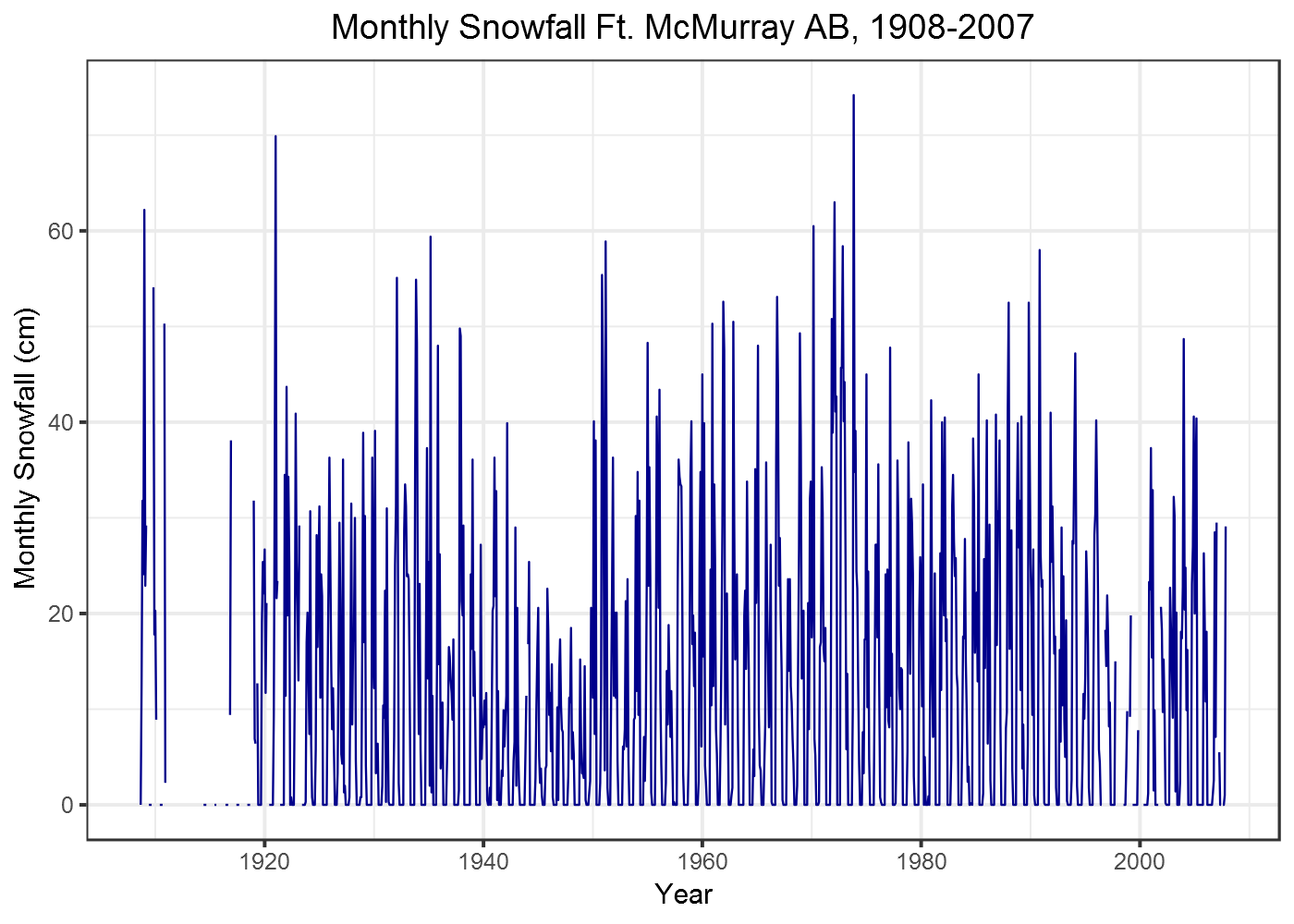
Figure S1.** Monthly snowfall 1908-2007 in Fort McMurray, Alberta, the city closest to our study area. There has been substantial variability of snowfall over the last century, but large snowfalls ( > 50 cm) have been more common in the past than in the last two decades. Data from Environment and Climate Change Canada, accessed October 2019.

**Figure S2.** Selection of generalized linear models (binomial errors, log link) of deer persistence (0-36 months) against natural and anthropogenic landscape features measured at multiple spatial scales. Stepwise analysis based on AIC scores shows features measured at the 1000-m radius spatial scale best explained white-tailed deer persistence (a) annually, and (b) in winter only. The weight of evidence in each case is high (AIC_w_ > 0.9), suggesting very strong support for this model.


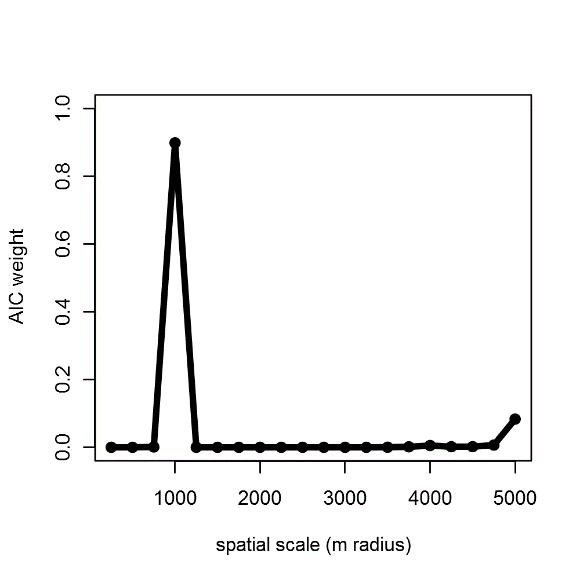

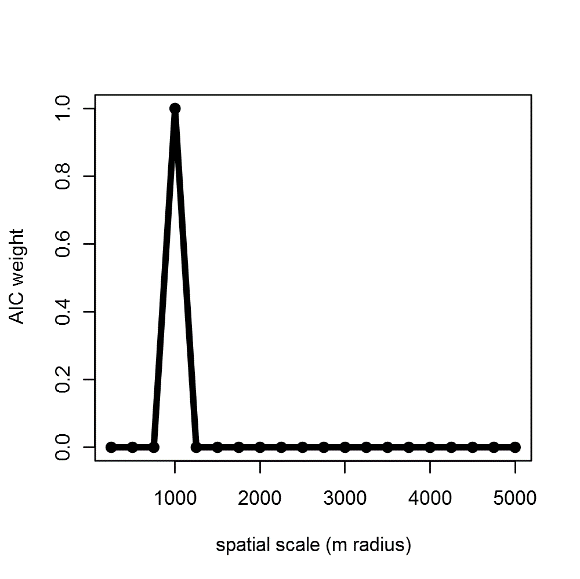


b

a

**Figure S3.** Model selection of generalized linear models (binomial errors, log link) of deer persistence against natural and anthropogenic landscape features measured at multiple spatial scales. Refer to Table S2 for constructs of the numbered models. (A) For annual deer persistence (0-36 months) model 28 was best supported of the *a priori* set. The *post hoc* reduced parsimonious model 31, including forestry, road, and petroleum features, best explained white-tailed deer persistence annually (AIC_w_ = 0.99). (A) For annual deer persistence (0-36 months) model 28 was best supported of the *a priori* set. The *post hoc* reduced parsimonious model 31, including forestry, road, and petroleum features, best explained white-tailed deer persistence annually (AIC_w_ = 0.99). (B) For winter deer persistence (0-9 months) model 28 was best supported of the *a priori* set. The *post hoc* reduced parsimonious model 31, including forestry, road, and petroleum features, best explained white-tailed deer persistence annually (AIC_w_ = 0.97).


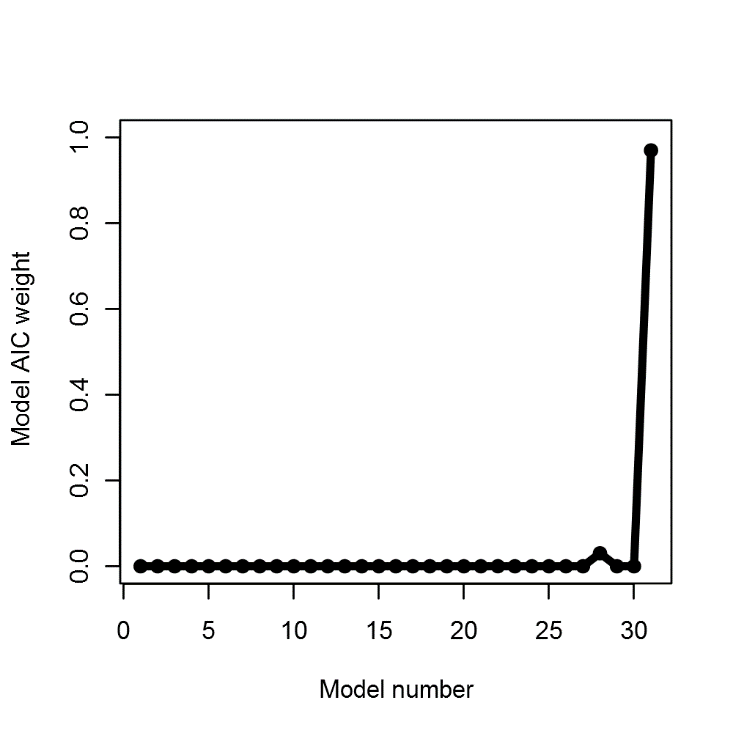

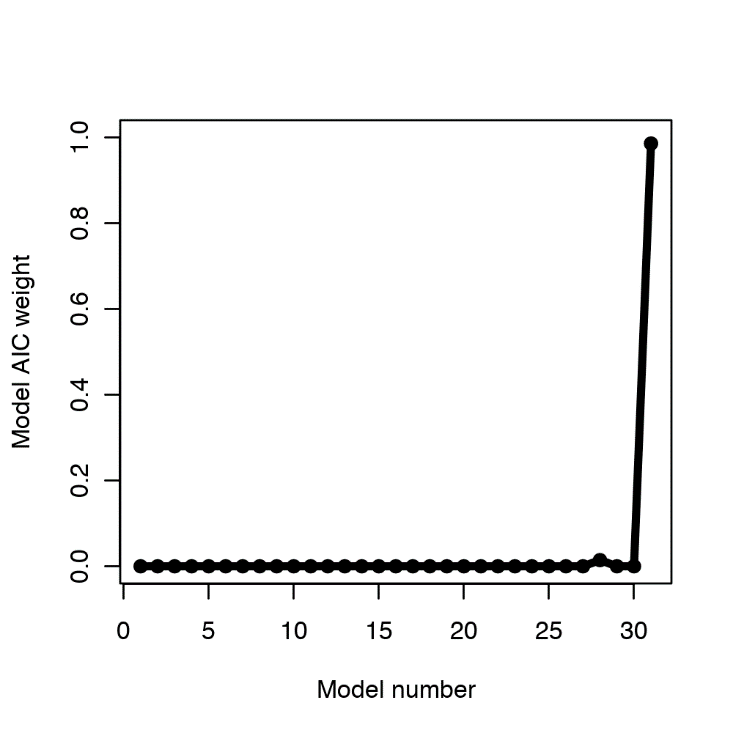


Annual

B

**Table S1.** Landscape reclassification for camera-based species distribution model analyses. GIS data from multiple sources were reclassified and combined to create 20 different landscape feature categories.

| Variable | Habitat Class | Source^1^ | Description^2^ |
| --- | --- | --- | --- |
| 1 | Upland deciduous | AVI | (Aw, Pb, Bw >=70% canopy), moisture = d or m |
| 2 | Lowland deciduous | AVI | (Aw, Pb, Bw >=70% canopy), moisture = w or a |
| 3 | Upland mixedwood | AVI | (40% -60%) canopy, moisture = d or m |
| 4 | Lowland mixedwood | AVI | (40% -60%) canopy, moisture = w or a |
| 5 | Upland spruce | AVI | (Sb, Sw, Fb >=70% canopy), moisture = d or m |
| 6 | Lowland spruce | AVI | (Sb,Sw,Fb >=70% canopy), moisture = w or a |
| 7 | Pine | AVI | All Pj (>=70%) |
| 8 | Tamarack | AVI | All Lt (>=70%) |
| 9 | Open wetland | AVI | <6% crown closure; moisture = w or a |
| 10 | Upland shrubs | AVI | >25% shrub cover; <6% tree cover; moisture=d or m |
| 11 | Water | AVI | Standing or flowing water |
| 12 | Cutblocks | ABMI | Forest harvested cutblocks of any age |
| 13 | Nonforest | AVI | Areas with < 6% canopy |
| 14 | Block features | ABMI | Combination variable including mining borrow pits, dugouts, sumps, industrial sites, and sites categorized as "other disturbed vegetation". No mature trees; usually reclaimed or surrounded by grass or shrubs. |
| 15 | Well sites | ABMI | Petroleum extraction sites including a well and surrounding area, usually grassy vegetation |
| 16 | 3D seismic lines | UALF | 3D seismic petroleum exploration lines, deployed in a high-density hashmark pattern. |
| 17 | Cutlines | UALF | Traditional, single petroleum exploration lines, less dense than 3D seismic lines. |
| 18 | Roads | UALF | Combination variable of one and two lane roads, gravel and paved, and unimproved roads. |
| 19 | Pipelines | UALF | Petroleum pipelines and their rights of way, usually wide and grass-covered. |
| 20 | Trails | UALF | Combination of trails (navigable by off road vehicle, horses, and people) and truck trails (navigable by trucks but without gravel). |

^1^AVI – Alberta Vegetation Index; UALF = University of Alberta Linear Features Map Updated 2012; ABMI = Alberta Biodiversity Monitoring Institute Human Footprint Map Updated 2010. ^2^Aw = aspen, Pb = poplar, Bw = white birch, Sb = black spruce Sw = white spruce, Fb = balsam fir, Pj = jack pine, Lt = tamarack/larch; d = dry, m = mesic, w = wet, a = aquatic

^3^Omitted from analysis to prevent collinearity with other variables.

^4^All linear features were buffered for areal calculations as follows: 'Two Lane Undivided Paved Road': 9m; 'One Lane Undivided Paved Road': 6m; 'Rail Line': 5.5m; 'Rail Line- spur': 5.5m; 'Two Lane Gravel Road': 7m; 'One Lane Gravel Road': 5m; 'Driveway': 2m; 'Unimproved Road': 6m; 'Trail': 6m; 'Truck Trail': 6m; 'Electrical Transmission Line': 17m; 'Pipeline': 12m; '3D': 2m; 'Cutline': 2m.

**Table S2.** *A priori* candidate models representing hypothesized relationships between white-tailed deer persistence and landscape features in the northeast boreal forest of Alberta, Canada.

| Description | Model # | Hypothesis:  WTD distribution is explained by: | Variables  (numbers refer to Table S1) |
| --- | --- | --- | --- |
| Global model | 1 | All variables | 1-20 |
| Natural landcover | 2 | Upland deciduous cover | 1 |
|  | 3 | All mixedwood cover | 3+4 |
|  | 4 | All conifer cover | 5+6+7+8 |
|  | 5 | Upland spruce | 5 |
|  | 6 | All deciduous + shrubs | 1+2+8+10 |
|  | 7 | Wetland | 9+11 |
|  | 8 | Upland forest | 1+3+5 |
|  | 9 | Lowland forest | 2+4+6 |
| Nonforest | 10 | Early seral | 10+12+13+14+15 |
| Forestry | 11 | Cutblocks | 12 |
| Petroleum | 12 | Wellsites | 15 |
|  | 13 | 3D seismic | 16 |
|  | 14 | Cutlines | 17 |
|  | 15 | Pipelines | 19 |
|  | 16 | Linear features | 16+17+18+19+20 |
|  | 17 | Block features | 14+15 |
| Petroleum + Forestry | 18 | Block features incl. cutblocks | 14+15+12 |
| Access | 19 | Roads | 18 |
|  | 20 | Trails | 20 |
|  | 21 | Roads and trails | 18+20 |
| All anthropogenic | 22 | All anthropogenic features | 12+14+15+16+17+18+19+20 |
| Natural + forestry | 23 | Upland deciduous and cutblocks | 1+12 |
|  | 24 | Shrubs and cutblocks | 10+12 |
|  | 25 | Openings and cutblocks | 10+12+13 |
| Natural + petroleum | 26 | Upland deciduous and 3D seismic | 1+16 |
|  | 27 | Upland deciduous and cutlines | 1+17 |
|  | 28 | Upland deciduous and all anthropogenic | 1+12+14+15+16+17+18+19+20 |
|  | 29 | Upland deciduous and all petroleum | 1+15+16+17+19 |
| Natural + access | 30 | Upland deciduous and roads and trails | 1+18+20 |
| Reduced *post-hoc* model | 31 | Upland deciduous and mixed anthropogenic | 1+12+14+15+20 |

**Table S3.** Multi-season occupancy models of deer detection-nondetection at cameras from October 2011 to October 2014. These models estimate the probability that a site is occupied (ψ), the probability that an empty site will be colonized (γ), and the probability that an occupied site will go “extinct” (ε). These parameters were either constant (c) or varied among 3-month SEASONs. The probability of detecting a white-tailed deer (if present), *p*, was either constant (c), varied in each SURVEY, or varied among SEASONs.

| Model | AIC | ΔAIC | AIC_w_ | ML^+^ | k* | -2LL** |
| --- | --- | --- | --- | --- | --- | --- |
| ψ,γ(SEASON),ε(SEASON),p(SURVEY) | 1210.29 | 0 | 1 | 1 | 39 | 1132.29 |
| ψ,γ(c),ε(SEASON),p(SURVEY) | 1244.90 | 34.61 | 0 | 0 | 33 | 1178.9.0 |
| ψ,γ(SEASON),ε(c),p(SURVEY) | 1246.57 | 36.28 | 0 | 0 | 33 | 1180.57 |
| ψ,γ(c),ε(c),p(SURVEY) | 1259.08 | 48.79 | 0 | 0 | 27 | 1205.08 |
| ψ,γ(SEASON),ε(SEASON),p(SEASON) | 1271.43 | 61.14 | 0 | 0 | 23 | 1225.43 |
| ψ,γ(SEASON),ε(c),p(SEASON) | 1305.30 | 95.01 | 0 | 0 | 17 | 1271.30 |
| ψ,γ(c),ε(SEASON),p(SEASON) | 1305.50 | 95.21 | 0 | 0 | 17 | 1271.50 |
| ψ,γ(c),ε(c),p(SEASON) | 1317.00 | 106.71 | 0 | 0 | 11 | 1295.00 |
| ψ,γ(SEASON),ε(SEASON),p(c) | 1347.99 | 137.70 | 0 | 0 | 16 | 1315.99 |
| ψ,γ(c),ε(SEASON),p(c) | 1384.73 | 174.44 | 0 | 0 | 10 | 1364.73 |
| ψ,γ(SEASON),ε(c),p(c) | 1410.47 | 200.18 | 0 | 0 | 10 | 1390.47 |
| ψ,γ(c),ε(c),p(c) | 1447.18 | 236.89 | 0 | 0 | 4 | 1439.18 |

^+^Model likelihood; *number of parameters; **-2 x log likelihood
